# Silicon Optical Modulator Using a Low-Loss Phase Shifter Based on a Multimode Interference Waveguide

**DOI:** 10.3390/mi10070482

**Published:** 2019-07-18

**Authors:** Daisuke Inoue, Tadashi Ichikawa, Akari Kawasaki, Tatsuya Yamashita

**Affiliations:** Toyota Central R&D Labs., Inc., 41-1, Yokomichi, Nagakute, Aichi 480-1192, Japan

**Keywords:** silicon photonics, modulator, multimode interferometer, photonics integrated circuit, carrier plasma, Mach–Zehnder interferometers

## Abstract

We have developed a novel phase modulator, based on fin-type electrodes placed at self-imaging positions of a silicon multimode interference (MMI) waveguide, which allows reduced scattering losses and relaxes the fabrication tolerance. The measured propagation losses and spectral bandwidth are 0.7 dB and 33 nm, respectively, on a 987 μm-long phase shifter. Owing to the self-imaging effect in the MMI waveguide, the wave-front expansion to the electrode was counteracted, and therefore, the scattering loss caused by electrode fins was successfully mitigated. As a proof-of-concept for the MMI-based phase modulator applications, we performed optical modulation based on Mach–Zehnder interferometers (MZIs). The π shift current of the modulator was 1.5 mA.

## 1. Introduction

Silicon (Si) photonics densely integrate optical devices, incorporating P–N junctions that enable the development of advanced devices, such as high-speed modulators, matrix switches, and variable attenuators [1,2,3,4,5,6,7].

One of the most common problems in Si photonics is the combination of channel waveguides and rib waveguides. Rib structures need large bending radii, which can cause large footprints. Combining a channel waveguide and rib waveguide is ideal for realizing more compact Si photonic circuits. However, a major issue in silicon photonics is the cost. Additional masks and processes are required to fabricate a rib structure [8]. 

Therefore, in this paper, we propose a novel optical phase modulator with a fin-type electrode based on a multimode interferometer (MMI) waveguide. It is noteworthy that our phase modulator has a wide working wavelength range. Furthermore, it can be fabricated at a low cost, because the minimum line width for processing the modulator is 0.4 μm.

## 2. Principle and Design

### 2.1. Princple

**Figure 1:**
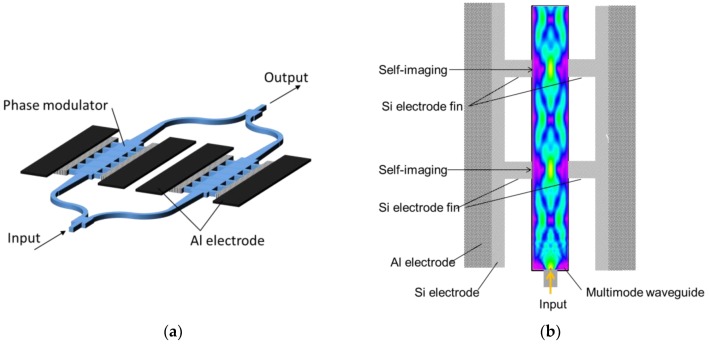
Schematic of (**a**) a Mach–Zehnder interferometer modulator with a multimode interferometer (MMI) phase modulator, and (**b**) a phase modulator based on MMI.

We used the carrier-injection method for fabricating a phase modulator composed of an MMI, as shown in Figure 1. The phase modulator is comprised of a multimode waveguide and is connected to a single-mode waveguide. The MMI causes periodic self-imaging of an input field profile [9].

As shown in Figure 1b, the electrode fins are placed at a distance where the self-imaging of an input field occurs. As a result, the scattering loss in the phase modulator caused by the electrode fins is reduced.

### 2.2. Design of the Phase Modulator

First, we determine the MMI length of the phase modulator via finite-difference time domain calculations under transverse electric-like polarization. The width of the MMI waveguide is ~1.2 μm, and the Si layer thickness is 0.2 μm, which provides a period length of 2.35 μm for self-imaging. A taper was inserted between the single-mode and multimode waveguides for reducing the connection loss. The length of the taper was 9 μm, and the width increased from 0.4 μm to 0.85 μm.

**Figure 2:**
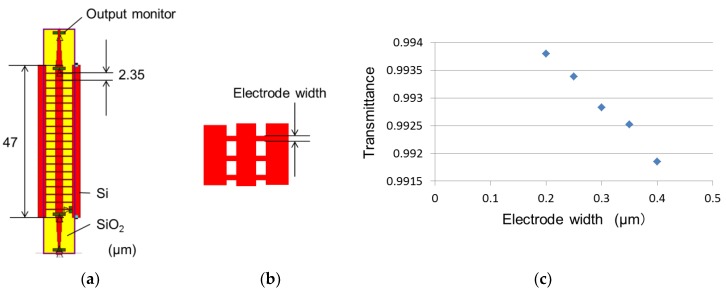
(**a**) Numerical calculation model of MMI phase modulator, (**b**) magnified drawing of electrode fin, and (**c**) transmittance (based on calculation results).

Next, we simulated propagation loss in the waveguide with electrode fins. Figure 2a shows the calculation model of the MMI phase modulator; the electrode width is defined in Figure 2b. Figure 2c shows the propagation loss in the multimode waveguide as a function of the electrode width. Figure 2c indicates that increasing the electrode width increases the propagation loss. However, the propagation loss in the waveguide with an electrode width of 0.4 μm is sufficiently thin for practical application. In addition to the propagation loss, the fabrication process was optimized for a line width of 0.4 μm. We fixed the width of the electrode fin to 0.4 μm for the above reasons.

### 2.3. Device Structure of the Optical Modulator

Our optical modulator is based on Mach–Zehnder interferometers (MZIs). We fabricated our MZI-based modulator using the MMI phase modulator with fin-type electrodes, as shown in Figure 1a. We fabricated two types of MZI modulators. One modulator had a different optical path, referred so as an asymmetric MZI. The other has the same optical path, called a symmetric MZI.

**Figure 3:**
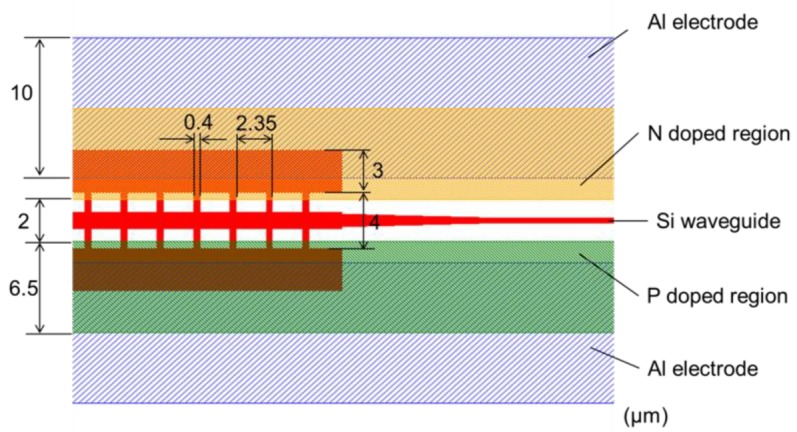
Layout of a phase modulator based on MMI.

Figure 3 shows the layout of the phase modulator based on an MMI. The length of the electrode fin is 1.4 μm. The P–N region at the side of the MMI phase modulator comprises highly doped P (4 × 10^15^ cm^−2^) and N (1 × 10^15^ cm^−2^) regions. This modulator only needs P and N regions. The gap between the P-type and N-type areas is 2 μm. A 0.5-μm-thick aluminum (Al) electrode is deposited on silicon (Si). The Si waveguide and Al electrode are covered with a 3-μm-thick oxide silicon layer and a 4-μm-thick SiO_2_ layer as the upper cladding layer. A 0.3-μm-thick tantalum layer was deposited on SiO_2_ as the heater. 

**Figure 4:**
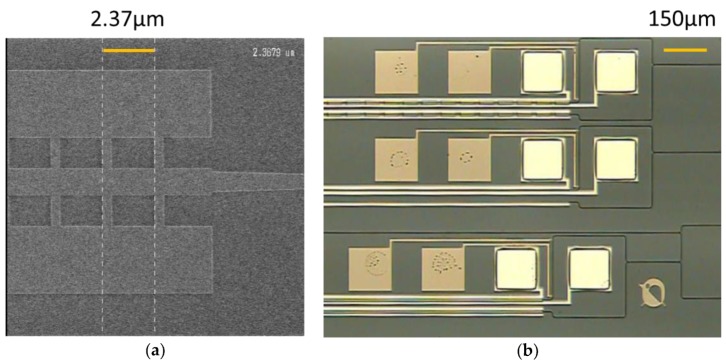
(**a**) Scanning electron microscope (SEM) image of an MMI waveguide with fins, and (**b**) an optical microscope image of a Mach–Zehnder interferometer (MZI) modulator formed using an MMI phase modulator. Orange lines indicate the scale.

Figure 4a shows the scanning electron microscope (SEM) images of the MMI phase modulator with fins before the deposition of the oxide silicon layer. Figure 4b shows the optical microscope image of the MZI-based modulator formed using this MMI phase modulator with electrode fins.

## 3. Experimental Results

### 3.1. Propagation Loss and Transmission Spectrum of a Multimode Interference Phase Shifter

**Figure 5:**
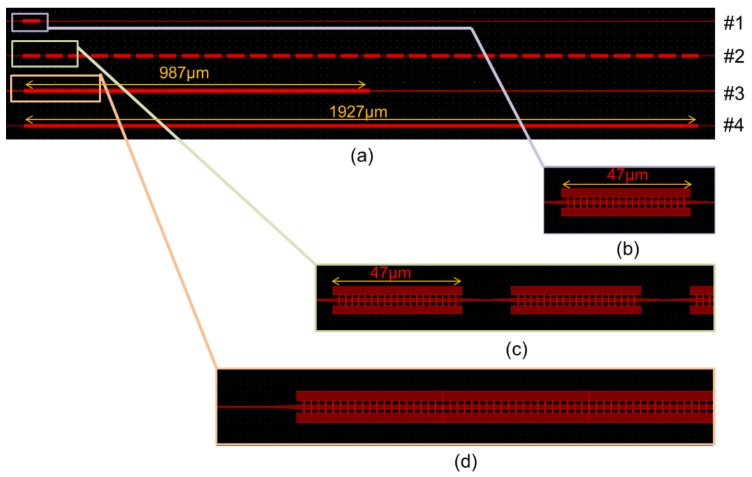
(**a**) Four types of MMI waveguides with fins for measuring propagation loss, and magnified drawing of (**b**) waveguide #1, (**c**) waveguide #2, and (**d**) waveguide #3.

We outsourced the fabrication of the photonics-integrated circuit. The foundry used electron beam lithography for the fabrication. The pitch here is about 0.4 μm, and we can achieve this with deep ultraviolet (UV) lithography, which has low cost for mass production. We fabricated four types of MMI waveguides with fins for measuring the propagation loss, as shown in Figure 5; in this figure, #1 represents 47 μm of the MMI waveguide length with fins, which is the same shape as was obtained via numerical calculation. Furthermore, #2 represents the 20 stages of cascade connections for 47 μm of the MMI waveguide length with fins. It is difficult to estimate small propagation losses in these samples. Therefore, we prepared #2 as a cascade connection of #1. In addition, #3 represents 987 μm of the MMI waveguide length with fins. Finally, #4 represents 1927 μm of the MMI waveguide length with fins. 

**Figure 6:**
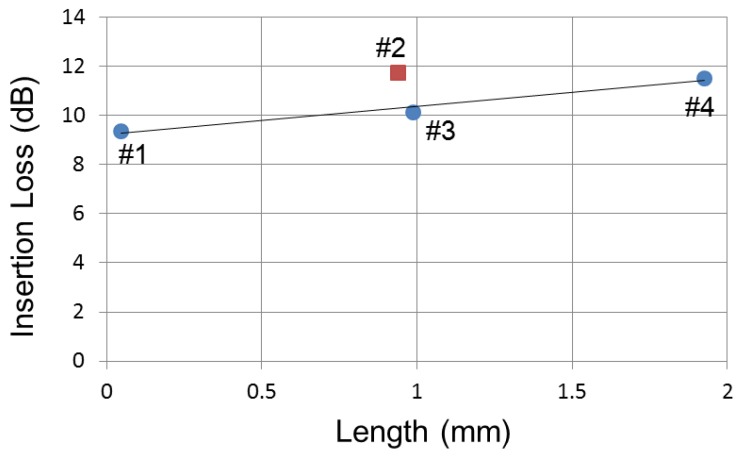
Length dependence of the insertion loss of an MMI waveguide with fins.

The light of the laser source is injected in the input facet of the device by butt coupling through a focused single mode fiber, and it is collected with a multimode fiber. The wavelength of the laser light was 1550 nm. For comparison, we measured the insertion loss in a single-mode waveguide with different lengths on the same wafer. To extract the propagation losses of the MMI waveguide, we drew a linear regression of the output power versus the length of devices 1, 3, and 4, as shown in Figure 6. We evaluated the insertion loss of an MMI waveguide with fins as 1.1 dB/mm from the coefficient of the result of the linear regression. The offset value of the linear regression of 9.2 dB, including the connection loss of a pair of tapers and the coupling loss at both the input and output ends between the spot size converter (SSC) and fiber. Assuming that the insertion losses and the propagation losses of the MMI waveguide are the same, we can predict the insertion loss of an MMI waveguide with 0.94 μm of length as 10.28 dB. However, the insertion loss of #2 was 11.74 dB, as shown in Figure 6. We consider that the difference is caused by the cascade connection of tapers. The differential number of tapers between waveguide #2 and #3 is 19 pairs. We estimated that the connection loss of a pair of tapers is 0.08 dB. The propagation loss in the straight waveguide on the same wafer was 0.32 dB/mm; there are 422 fins per 1 mm of MMI waveguide. We evaluated the scattering loss of a fin as 0.0018 dB. We considered that the insertion loss of the MMI waveguide with fins is considered sufficient for practical use. 

**Figure 7:**
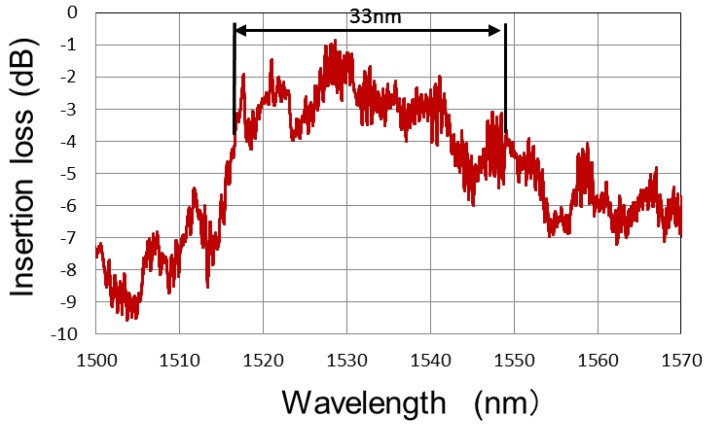
Transmission spectrum of an MMI waveguide with fins.

We measured the transmission spectrum for 987 μm of the MMI waveguide length with fins. We input light waves emitted from a super-luminescent laser diode (SLD) into the MMI waveguide with fins with a tapered fiber, and measured the transmission spectrum using an optical spectrum analyzer (OSA). As a reference, we measured the spectrum of the SLD. Figure 7 shows the transmission spectrum of the MMI waveguide with fins. The full width at half maximum (FWHM) in the transmission spectrum of the MMI waveguide with fins was 33 nm. We decided that 987 μm of length would be appropriate for phase modulators, owing to the aforementioned reason.

### 3.2. Shift in Transmission Spectrum and Modulation by Current Injection

**Figure 8:**
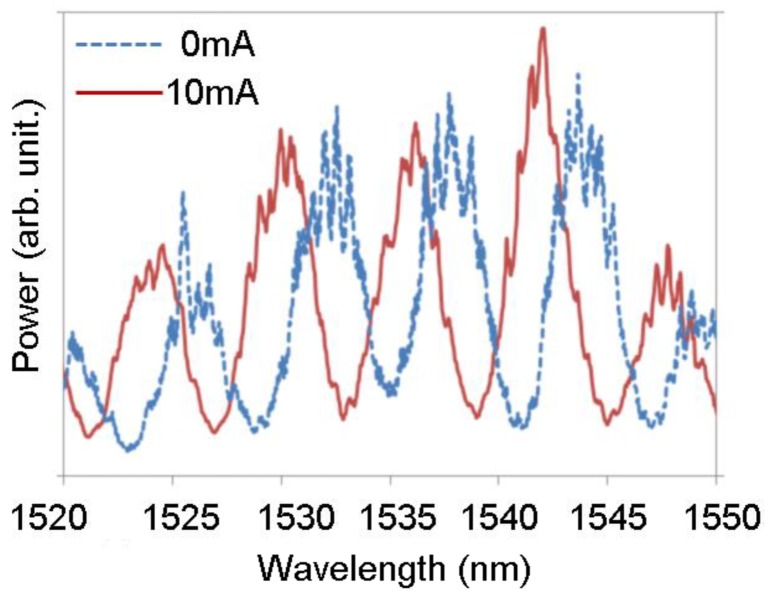
Shift in transmission spectrum of an MZI-based modulator with an MMI phase modulator caused by current injection.

First, we measured the shift in the transmission spectrum of the asymmetric MZI modulator. We contacted the P and N electrodes with needle probes and injected the forward bias current. We input light waves delivered from the SLD into the MZI modulator with a tapered fiber, and measured the transmission spectrum with the OSA. Figure 8 shows the output spectrum of the MZI modulator under injection currents of 0 mA and 10 mA. On increasing the injection current, the peaks shifted toward shorter wavelengths. When the carrier plasma effect occurs, increasing the carrier density decreases the refractive index. On the other hand, increasing the temperature will increase the refractive index. We confirmed that this wavelength shift was caused by the carrier plasma effect.

**Figure 9:**
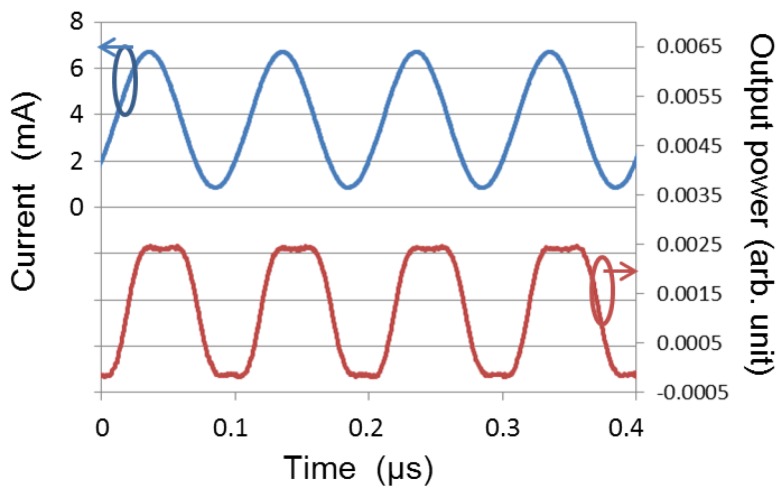
Output power of an MZI modulator with MMI phase modulator driven by alternating current (AC) power.

**Figure 10:**
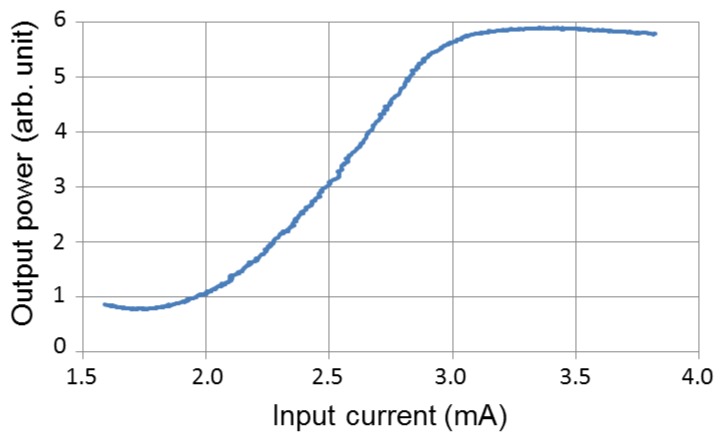
Injection current dependence of output power of an MZI modulator with an MMI phase modulator.

Next, we attempted to modulate light waves. We input laser light into the symmetric MZI modulator with a tapered fiber and coupled the output light with a multimode fiber. The wavelength of the light was 1550 nm. We applied alternating current (AC) at 10 MHz to the MZI modulator. The optical output was detected using an optical/electrical converter (1444-50, Newport, Irvine, CA, USA). Electrical signals were captured by using a sampling oscilloscope (MSO4104, Agilent, Santa Clara, CA, USA). Figure 9 and Figure 10 show the measurement results of the modulated light waves. We measured output power, as a function of the injection current. It appears that the injection current reached the half-wave current, because both the ceiling and floor of the waveforms were distorted.

## 4. Discussion

Here, we discuss the issues pertaining to MMI phase shifters. When light passes through an MMI structure, internal reflection occurs. In our experiment, we observed some ripples, which are indicated in the transmission spectrum shown in Figure 8.

The free spectrum range (FSR) of a Fabry–Perot resonator is expressed as

$$\mathrm{FSR}=\frac{\lambda^{2}}{2n_{r}l},$$

where *λ* is the wavelength, and *n_r_* and *l* represent the refractive index and length of the cavity, respectively. The period of the ripples in the transmission spectrum of the MMI waveguide shown in Figure 8 is 0.55 nm; the measurement wavelength is 1.55 μm. The length of the phase shifter is 987 μm. The refractive index of the silicon waveguide was calculated to be 2.1. Since not one mode but a few modes propagate through an MMI waveguide, we calculated the FSR of the MMI waveguide with an average refractive index; the FSR was predicted to be 0.57 nm. The calculated FSR agrees with the period of the ripples in the transmission spectrum.

However, the transmission spectrum of the MMI waveguide shown in Figure 7 has two types of ripples. The period for one type of ripple is 5 nm, and that for the other type is 0.55 nm. The ripple with a 0.55 nm period was attributed to the internal reflection in the MMI waveguide. The ripple with a 5 nm period corresponds to 11 μm of cavity length. The length of one of the tapers is 9 μm, while the other is close to 11 μm. The ripple with a 5 nm period is considered to be caused by a taper.

The period of the fins in the MMI phase shifter was 2.35 μm. The FSR for a period of 2.35 μm is expected to be 24 nm. However, 24 nm ripple periods are not observed in either Figure 7 or Figure 8. Hence, we assume the design of the fins to be satisfactory.

## 5. Conclusions

We fabricated and demonstrated a phase-shifting structure suitable for silicon photonics circuits. A phase-shifter based on an MMI was constructed from multimode waveguides and electrode fins placed at a distance where the self-imaging of an input field occurs. Self-imaging at the center of the MMI-based phase shifter counteracted the wave-front expansion, thus mitigating the scattering loss caused by the electrode fins.

The proposed phase modulator does not need the half-etching process. The minimum linewidth was 0.4 μm, and a low-cost lithography process was sufficient for this modulator. In addition to the low minimum line width, this phase modulator required only P+ and N+ regions. Therefore, the fabrication process was simplified, and the costs were reduced.

We fabricated MMI-based multimode waveguides with fins and evaluated the insertion loss into them. The propagation loss for 987 μm of the MMI waveguide length with fins was 0.7 dB higher than that for the same length of a single-mode waveguide. The FWHM in the transmission spectrum of the phase shifter with electrode fins was 33 nm. The propagation loss was small enough, and the FWHM in the transmission spectrum of the phase-shifter with electrode fins was wide enough for practical applications.

We injected current into the fabricated modulator and measured the change in the transmission spectrum. The shift in the spectrum indicates that the working of this modulator is governed by the carrier plasma effect. We demonstrated the modulation of light waves by applying AC to the modulator.
